# Effects of school-based active breaks on motor competence, physical fitness, academic outcomes, and health behaviors in Chilean preschoolers: protocol for a three-arm, non-randomized cluster-controlled mixed-methods trial

**DOI:** 10.3389/fpubh.2026.1895830

**Published:** 2026-09-23

**Authors:** María Alejandra Valencia-Pacheco, Viviana Vallejos Garcías, Karen Kracht-Suazo, Daniela Gallegos Abello, Nicole Lassere Laso, Alejandra Robles-Campos, Daniel Reyes-Molina, Igor Cigarroa, Johana Soto-Sánchez, Jorge Rojas-Bravo, Carolina Muñoz Pérez, Jessica Ibarra-Mora, Tomás Reyes-Amigo, Sonia Sepúlveda-Martín, Pamela Muñoz-Ramos, Javier Russell-Guzmán, Rafael Zapata-Lamana, Alberto Ruiz-Ariza, Natalia María Arias Palencia

**Affiliations:** 1 Escuela de Educación, Universidad de Concepción, Los Ángeles, Chile; 2 Facultad de Educación, Universidad de Concepción, Concepción, Chile; 3 Escuela de Kinesiología, Facultad de Salud, Universidad Santo Tomás, Los Ángeles, Chile; 4 Dirección Comunal de Educación, Los Ángeles, Chile; 5 Escuela de Nutrición y Dietética, Facultad de Salud, Universidad Santo Tomás, Los Ángeles, Chile; 6 Departamento de Psicología, Universidad de Concepción, Concepción, Chile; 7 Escuela de Kinesiología y Terapia Ocupacional, Facultad de Ciencias de la Salud, Universidad Católica Silva Henríquez, Santiago, Chile; 8 Facultad de Ciencias de la Salud, Universidad Arturo Prat, Victoria, Chile; 9 Biomedicine Center, Laboratory of Physical Activity, Exercise and Health, Faculty of Medicine and Health Sciences, Universidad Mayor, Santiago, Chile; 10 Departamento de Educación Física, Deportes y Recreación, Universidad Metropolitana de Ciencias de la Educación, Santiago, Chile; 11 Physical Activity Sciences Observatory (OCAF), Universidad de Playa Ancha, Playa Ancha, Región de Valparaíso, Chile; 12 Departamento de Ciencias Clínicas y Preclínicas, Facultad de Medicina, Universidad Católica de la Santísima Concepción, Concepción, Chile; 13 Liceo Alemán, Los Ángeles, Chile; 14 School of Kinesiology, Faculty of Health, Universidad Santo Tomás, Santiago, Chile; 15 Physical Activity and Sport Sciences, Faculty of Health, Universidad SEK, Santiago, Chile; 16 Centro de Vida Saludable, Universidad de Concepción, Concepción, Chile; 17 Faculty of Educational Sciences, University of Jaen, Jaén, Spain; 18 Faculty of Education of Cuenca, University of Castilla-La Mancha, Cuenca, Spain

**Keywords:** active breaks, health behavior, mixed methods, motor competence, physical fitness, preschool, school-based intervention

## Abstract

**Background:**

Although school-based physical activity (PA) is associated with improvements in multiple health and learning indicators, significant gaps persist regarding its systematic implementation at the preschool level. This protocol presents the rationale and methodological design of a curriculum-integrated active break program aimed at evaluating its impact in 5- to 6-year-old preschoolers.

**Methods:**

A three-arm, non-randomized cluster-controlled trial employing a sequential explanatory mixed-methods design (QUAN → qual) will be conducted in 12 urban public schools in the province of Biobío, Chile, involving 200 kindergarten preschoolers. Primary outcomes—motor competence and health-related physical fitness—and secondary outcomes—learning outcomes and healthy habits (sleep, eating behavior, nutritional status)—will be assessed at baseline and post-intervention. Over 16 weeks, children in the Total Experimental Group (TEG; 4 schools, *n* = 67) will complete 10 active breaks per week; the Partial Experimental Group (PEG; 3 schools, *n* = 66) will complete 3–5 active breaks per week; and the Control Group (CG; 5 schools, *n* = 67) will continue regular daily routines.

**Discussion:**

To the best of our knowledge, this is the first controlled trial to simultaneously examine the efficacy of a curriculum-integrated active break program on motor competence, physical fitness, learning outcomes, and healthy habits in preschool-aged children. The inclusive design, digital platform support, and mixed-methods approach position this trial as a novel contribution to the evidence base for early childhood PA interventions and educational public policy.

**Study protocol registration:**

Open Science Framework (OSF). Unique identifier: cu6za. Public URL: https://osf.io/cu6za/overview.

## 1 Introduction

A global consensus exists regarding the low levels of physical activity (PA) among the preschool population (1). Reports from the World Health Organization (2) and the Global Matrix 4.0 (2022) indicate that more than 80% of children over 5 years of age do not meet the internationally recommended 60 min of moderate-to-vigorous PA per day (3). This PA insufficiency, combined with increasing sedentary behavior, constitutes a growing public health problem with serious short- and long-term consequences (4).

Recent research links low PA levels with poor academic performance, impaired socioemotional well-being, sleep disturbances, and excess malnutrition (2). Negative effects on physical, mental, and social dimensions have also been reported (5). In contrast, regular PA—together with adequate sleep hygiene—is associated with stronger physical and mental health trajectories over time (6, 7). Higher PA levels in childhood are also linked to more self-determined eating patterns, constituting a set of key wellness habits (8).

The literature consistently supports the benefits of PA for health and for the development of physical, academic, and cognitive capacities (9). Recent evidence further suggests that even brief interventions can have a significant health impact (10): as few as 5 min of daily PA may prevent up to 6% of mortality in high-risk groups and 10% in a population-based approach (11).

In this context, the implementation of PA intrinsic to the school curriculum emerges as a promising strategy (2, 12). Educational settings are strategic spaces, given that students spend the majority of their waking hours there during key developmental stages. Within this environment, inclusion must serve as the organizing axis for addressing student diversity (13), ensuring that PA strategies reach all students.

Based on the Active Schools model (14), several PA strategies have been proposed for the educational context, including active recess, physically active lessons, quality physical education, active transportation, and active breaks (15). Active breaks consist of brief periods of physical exercise integrated into the school day (16, 17). Evidence from systematic reviews reports positive outcomes for PA levels, behavior, and executive functions (18–20), reductions in sedentary time (21), and improvements in cognitive variables—attention, working memory, inhibitory control, and cognitive flexibility (22, 23), as well as classroom behavior (9, 10), cognitive efficiency (24, 25), and physical and psychological outcomes (26, 27). The integration of active breaks with curricular content positions them as viable and effective strategies adaptable to curricular needs (28).

However, significant gaps persist. Further research is needed on intervention strategies and their impact on the didactic process, particularly in the preschool population (16, 29). Evidence for children aged 5–6 years is scarce, and even more limited for students with Special Educational Needs (SEN) (30). In this age group, limited studies have reported positive impacts on cognitive engagement, motor competence, attention, and emotional regulation (10, 31). Only one systematic review has addressed active breaks in children as young as 5 years (9), underscoring the need for interventions that guarantee effective participation by all students.

Internationally, several countries have established legal frameworks to promote PA in educational settings (32–36), reinforcing the relevance of such interventions for public policy. Despite these advances, no controlled trial has specifically evaluated the efficacy of curriculum-integrated active breaks in preschool children aged 5–6 years. The present protocol addresses this gap by describing the rationale and methodological design of a trial evaluating the efficacy of active breaks on motor competence, physical fitness, academic performance, and healthy habits in Chilean preschoolers.

## 2 Methods

### 2.1 Study design

This protocol employs a three-arm, non-randomized cluster-controlled trial using a sequential explanatory mixed-methods design (DEXPLIS), (37), with single-blind evaluation: assessors and data analysts are blinded to group allocation. The study was pre-registered in the Open Science Framework (OSF), (38) and will adhere to the TREND guidelines (Transparent Reporting of Evaluations with Non-Randomized Designs; Supplementary material 1). Table 1 summarizes the study design.

**Table 1:** Study design summary.

Design element	Specification
Study type	Non-randomized, cluster-controlled trial
Mixed-methods design	Sequential explanatory (DEXPLIS)—QUAN → qual
Number of arms	Three: Total Experimental Group (TEG), Partial Experimental Group (PEG), Control Group (CG)
Allocation unit	School (cluster)
Blinding	Single-blind: evaluators and data analysts are blinded to group allocation
Primary outcomes	Motor competence (MOBAK-KG); health-related physical fitness (PREFIT)
Secondary outcomes	Learning outcomes (DIA EP); sleep (BEARS); eating behavior (CEBQ); nutritional status/cardiometabolic risk (anthropometry)
Intervention duration	16 weeks
Assessment points	Baseline (weeks 1–4) and post-intervention (weeks 23–26)
Setting	12 urban municipal schools, Los Ángeles, Biobío Region, Chile
Pre-registration	Open Science Framework (OSF)
Reporting guideline	TREND (Transparent Reporting of Evaluations with Non-Randomized Designs)

### 2.2 Study context

Seventeen urban public schools from the commune of Los Ángeles, province of Biobío, Chile—representing 100% of the establishments providing kindergarten education for 5- to 6-year-old children—were invited to participate. Schools were selected for three reasons: (1) Los Ángeles is the provincial capital with the highest level of urbanization in the Province of Biobío; (2) it has the highest probability of meeting the material conditions required by the project, particularly connectivity and minimum equipment for platform use; and (3) it offers logistical proximity due to the presence of higher education institutions. Schools share similar School Vulnerability Index (SVI) levels (39) and are located in lower-middle socioeconomic contexts, operating an approximately 8-h school day (08:00–16:00) with four 15-min recesses (09:30, 11:00, 12:45, and 15:15), a breakfast break (09:00–09:30), a lunch break (12:00–12:30), and an afternoon snack break (14:30–15:00).

The selection framework was developed in coordination with the Municipal Education Administration Department (DAEM). From the 17 eligible schools, 12 accepted participation and constitute the final study sample. Random allocation was not feasible given ethical and operational constraints; schools were therefore assigned to groups through purposive criteria (see Section 2.4). Figure 1 (see Supplementary material) presents the flow chart of participants through the study.

**Figure 1:**
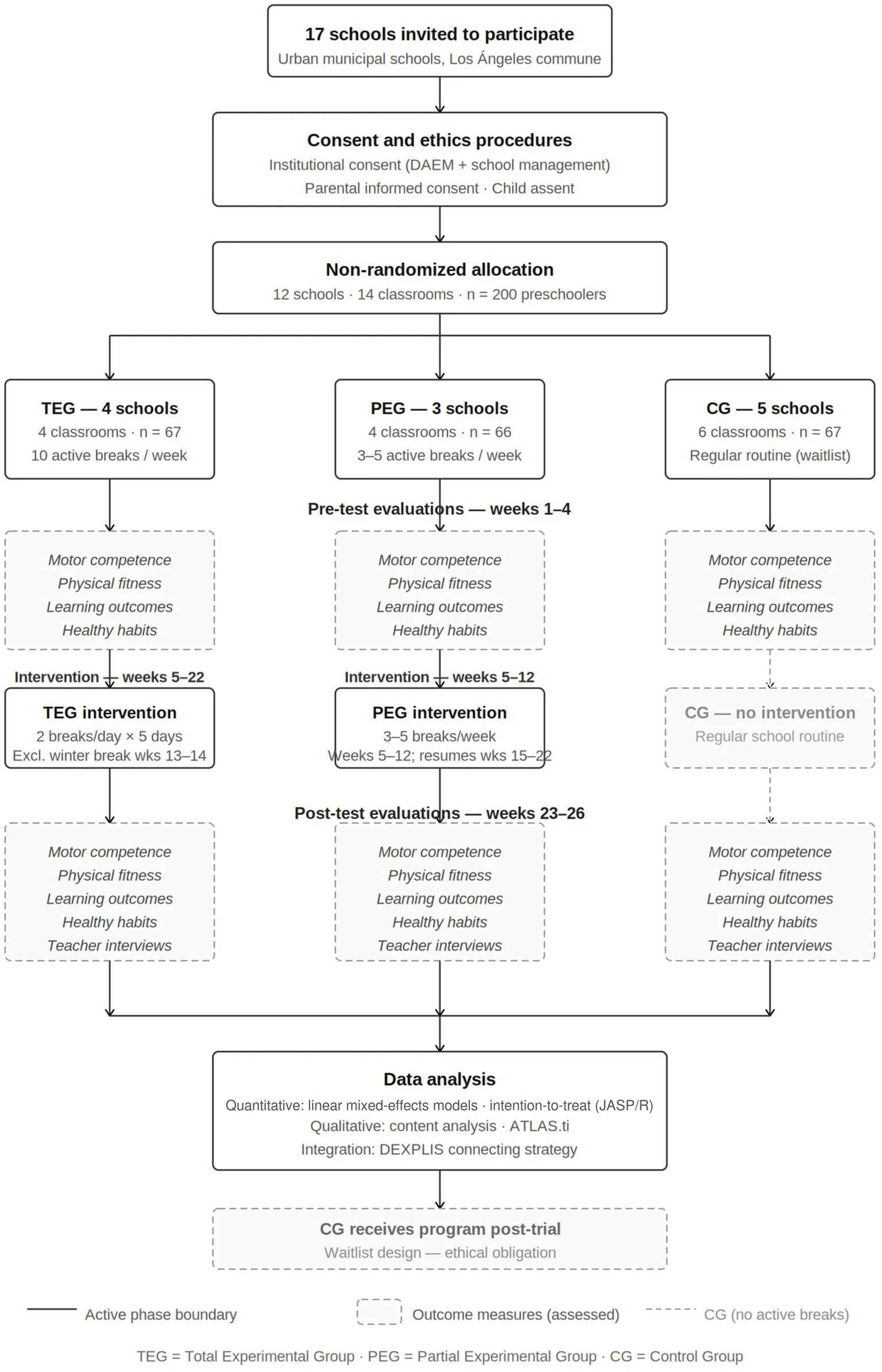
Flow chart of participants through the Active Kindergarten Project (AKP). TEG, Total Experimental Group; PEG, Partial Experimental Group; CG, Control Group. Dashed boxes indicate outcome measures assessed at each time point. The CG operates as a waitlist: Schools receive the active break program after trial completion.

### 2.3 Sample size

Sample size was estimated *a priori* using G*Power software, with motor competence (MOBAK-KG) as the primary outcome. A moderate-to-large effect size (*f* = 0.25–0.40) was assumed based on a meta-analysis of PA interventions targeting physical fitness development in preschool populations (40). A significance level of *α* = 0.05 and 80% power were set for three-group comparisons. Given the cluster design (schools), sample size was adjusted using the design effect DE = 1 + (*m* − 1) × ICC, where ICC = 0.05, consistent with typical values in school-based preschool intervention studies (41). With 12 schools, an average cluster size of 16.7 students, and ICC = 0.05, a DE of 1.79 was estimated, yielding an effective sample size of approximately 112 independent observations. A total sample of 200 preschoolers (approximately 67 per group) was planned, accounting for clustering effects and an anticipated 10–15% attrition rate.

### 2.4 Recruitment and school allocation

Recruitment began with initial email communication and telephone calls to the Municipal Education Directorate (DAEM), based on the MINEDUC Ministry of Education database (42). Schools that agreed to participate received a project description in accessible language and an authorization form. Once institutional endorsement was formalized, researchers visited schools to present the study and obtain support from management and teaching staff. The proposal was then presented to families at the first annual parent meeting, where questions were addressed and informed consent for children’s participation was collected. As the final ethical step, assent was obtained from each preschooler before any data collection.

### 2.5 Eligibility criteria

Of the 17 eligible schools, 12 agreed to participate. Following acceptance, schools were assigned to one of three groups based on the following institutional criteria: (i) availability of a computer and digital projection screen; (ii) explicit willingness of the kindergarten teacher to participate; and (iii) teacher use of the DIA EP integrated learning diagnostic platform. Table 2 presents the final group allocation, and Table 3 summarizes inclusion and exclusion criteria at both school and individual levels.

**Table 2:** Group allocation: schools, classrooms, and students.

Group	Schools (*n*)	Classrooms (*n*)	Students (*n*)	Active breaks/Week	Duration per break
Total experimental group (TEG)	4	4	67	10 (2/day × 5 days)	6–8 min
Partial experimental group (PEG)	3	4	66	3–5 (variable)	6–8 min
Control group (CG)	5	6	67	0 (waitlist)	—
Total	12	14	200		

Schools: 4 TEG + 3 PEG + 5 CG = 12. Classrooms: 4 TEG + 4 PEG + 6 CG = 14 (PEG includes one school with two kindergarten classrooms). All numbers are pre-intervention planned allocations.

**Table 3:** Inclusion and exclusion criteria.

Level	Inclusion criteria	Exclusion criteria
School	(1) Urban municipal school offering kindergarten; (2) sufficient kindergarten enrollment; (3) basic technological infrastructure (connectivity + projection screen); (4) kindergarten teacher willing to participate; (5) use of DIA EP diagnostic platform	Schools not meeting any inclusion criterion; private or subsidized private schools
Individual (child)	(1) Enrolled in kindergarten (age 5–6); (2) ≥ 85% attendance during implementation period; (3) parental/guardian consent; (4) child assent. Children with and without SEN are eligible	(1) Insufficient Spanish proficiency to understand instructions (teacher-assessed); (2) physical or mental health condition preventing participation (reported by teacher, parent, or guardian); (3) diagnosed chronic disorder restricting PA (cardiac disease, diabetes, asthma, musculoskeletal injury)

### 2.6 Participants

The study population comprises preschoolers aged 5–6 years enrolled in urban public schools in the commune of Los Ángeles, Biobío Region, Chile. The study inclusively considers the participation of preschoolers with and without SEN. The sample of 200 preschoolers was obtained through non-probabilistic convenience sampling across 14 classrooms from 12 urban public schools, with prior authorization from the DAEM. For ethical reasons, the Control Group will be treated as a waitlist: CG schools will receive the active break program after the experimental group intervention concludes.

## 3 Intervention protocol

### 3.1 Pilot phase

Prior to the main study, a pilot phase was conducted in a school outside the study sample, with children aged 5–6 years, to test and adjust research procedures in a controlled, small-scale setting. Adjustments included: active break duration; children’s comprehension of instructions and questions; response interpretation; complexity level; exercise sequences and execution; number of participants; and material resources (audio, images, classroom space). A safety log was maintained to record and monitor accident risks during sessions (Supplementary material 2).

### 3.2 Technological component

The study uses the interactive web platform Clases Activas^1^, a previously validated educational resource (43). The platform provides a repository of validated exercises designed to improve students’ coordination and fundamental motor skills, including bilateral body coordination exercises (lateral jumps, rope jumping, forward/backward jumps, jumping jacks), fundamental motor skill tasks (balance and locomotion), and muscular strengthening exercises (squats).

The platform features three access profiles: (i) Administrator—research team; manages schools, monitors usage, creates challenges, and analyzes data. (ii) School climate coordinator—one professional per school; manages teacher registration and monitors challenge progress. (iii) Teacher—creates and implements active breaks in the classroom and tracks progress. Clases Activas incorporates a gender-equity perspective and promotes cooperative learning through interactive exercises featuring two animated characters, Paz and Ayun, who verbalize questions and response options. An accessible resources section provides exercises adapted for students with limited mobility. Each teacher has a personalized account. The program requires no specialized PA knowledge, enabling generalist teachers and preschool educators to serve as effective facilitators (43).

### 3.3 Curricular component: curriculum-based active breaks

Chile’s Early Childhood Education Curriculum Guidelines (BCEP) constitute the central normative and pedagogical reference for ages 0–6, organized around three components: learning domains, learning nuclei, and levels or stages. In Clases Activas, kindergarten teachers implement active breaks aligned with the academic content and learning objectives of three nuclei: verbal language, mathematical thinking, and coexistence and citizenship. One learning objective from one nucleus is selected per session; each active break contains eight questions with two response alternatives per question (Figure 2; Supplementary material 3a).

**Figure 2:**
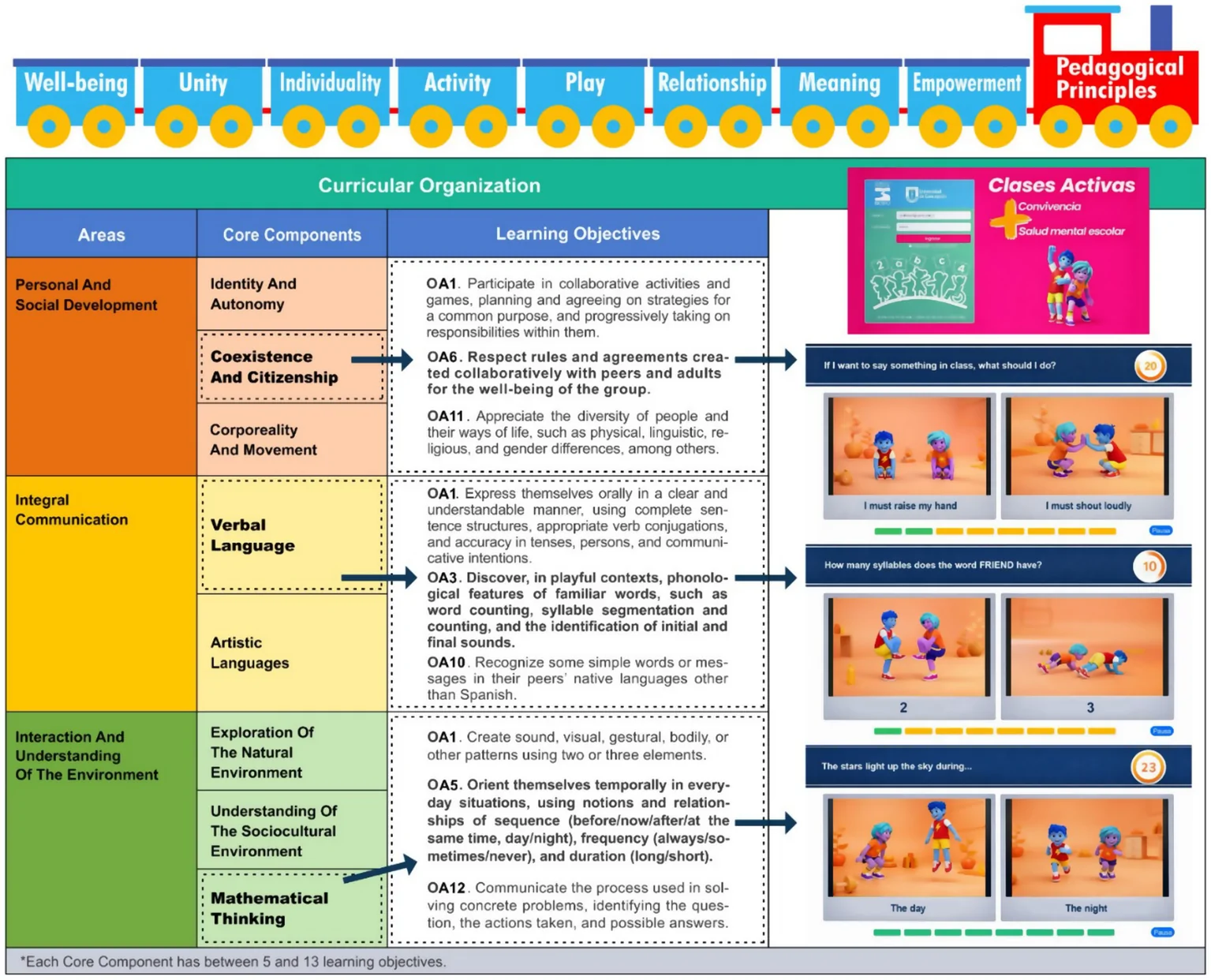
Integration of curriculum learning objectives with the Clases Activas platform. The left panel shows the BCEP curricular structure (domains, nuclei, learning objectives) linked to the three targeted learning domains. The right panel shows the platform interface and exercise examples with the animated characters Paz and Ayun. Source: Authors’ own elaboration using the Clases Activas platform.

Session scheduling will be integrated into school timetables as a natural interruption in the daily routine, particularly when children: (i) have been seated for extended periods; (ii) are not paying attention; or (iii) are fatigued. The three nuclei are practiced in rotating alternating order (Supplementary material 3b). Each active break consists of three phases:

(1) Welcome and preparation: children stand with sufficient space for in-place exercises; the platform is projected; each break opens with a motivational message from Paz and Ayun.
(2) Main content: execution through the 8 questions; includes exercises adapted for students with limited mobility.
(3) Closing and cool-down: stretching and a final motivational message from the characters.

This program draws on prior work by our research group (23, 43).

## 4 Variables and instruments

Table 4 provides a summary of all variables, instruments, administrators, and assessment points.

**Table 4:** Summary of variables, instruments, and assessment schedule.

Variable	Type	Instrument	Respondent/Administrator	Assessment point	Format
Motor competence	Primary	MOBAK-KG (44)	Trained evaluators (kinesiology students)	Baseline + post	Objective test
Health-related physical fitness	Primary	PREFIT battery (48, 49)	Trained evaluators (kinesiology students)	Baseline + post	Objective test
Learning outcomes	Secondary	DIA EP rubrics—verbal language, math thinking, coexistence & citizenship (53)	Kindergarten teacher	Baseline + post	Rubric
Sleep	Secondary	BEARS Sleep Screening Tool (54)	Parent/guardian	Baseline + post	Questionnaire (5 min)
Eating behavior	Secondary	Child Eating Behavior Questionnaire—CEBQ (55, 56)	Parent/guardian	Baseline + post	Questionnaire (10 min)
Nutritional status	Secondary	BMI-for-age; WC-for-age; WHtR (60)	Trained evaluators (nutrition students)	Baseline + post	Anthropometry (10 min)
Teacher perceptions	Secondary/Qualitative	Semi-structured interview—phenomenological design (62)	Research team	Post-intervention	Audio-recorded interview
Sociodemographic data	Covariate	Custom 33-item form (Supplementary material 5)	Parent/guardian	Baseline only	Questionnaire (10 min)

### 4.1 Primary variables

#### 4.1.1 Motor competence

Motor competence will be assessed using the MOBAK-KG battery (44), widely used internationally and validated for high reproducibility (45, 46). The MOBAK-KG assesses children aged 4–6 years across two dimensions: Body Control (balancing, rolling, jumping, running) and Object Control (throwing, catching, hand dribbling, foot dribbling), each comprising four tasks. Scoring uses a standardized dichotomous system (0–2 per task), yielding a maximum of 8 points per dimension, enabling differentiated interpretation of motor development levels. Content validity was established in alignment with curriculum guidelines; construct validity and reliability were confirmed through confirmatory factor analysis (*n* = 403), (45). A recent study validated the MOBAK-KG for preschool settings in Colombia (47). The battery is applicable for screening, diagnosis, monitoring, group characterization, and intervention evaluation.

All tests will be conducted in the school yard or gymnasium during school hours, in small groups of six participants per station. A rest period of 3–5 min will be provided between tests to mitigate fatigue and sequencing effects. Estimated total duration: 35 min.

#### 4.1.2 Health-related physical fitness

Health-related physical fitness will be assessed using the PREFIT battery (48), validated for children aged 3–5 years and recently validated for moderate-to-excellent reliability in Chilean preschoolers aged 4–6 years (49). Components include cardiorespiratory capacity, musculoskeletal capacity, motor capacity, and body composition, measured via BMI, waist circumference, handgrip strength, standing broad jump, speed-agility, and 20-m shuttle run (50–52).

Both MOBAK-KG and PREFIT will be administered by pre-trained kinesiology and special education students, supervised by a researcher (Supplementary material 4).

### 4.2 Secondary variables

#### 4.2.1 Learning outcomes

Learning outcomes will be assessed using the DIA EP rubrics (Diagnóstico Integral de Aprendizajes en Educación Parvularia), (53), a formative assessment tool available from MINEDUC for kindergarten and pre-kindergarten levels. The tool covers three learning nuclei: verbal language, mathematical thinking, and coexistence and citizenship. Progress is categorized across four qualitative levels: beginning, developing, consolidating, and deepening. Although designed primarily as a formative tool, it provides a nationally standardized framework for comparing progress across groups.

#### 4.2.2 Sleep

Sleep will be assessed using the BEARS Sleep Screening Tool, designed to identify possible sleep disorders in children and adolescents aged 2–18 years across five dimensions: bedtime issues, excessive daytime sleepiness, night awakenings, regularity/duration, and snoring. The instrument has demonstrated adequate reliability and validity in Colombian school-age children and adolescents (54). Parents or guardians will complete the questionnaire at a parent meeting or online (estimated time: 5 min).

#### 4.2.3 Eating behavior

Eating behavior will be assessed using the Child Eating Behavior Questionnaire (CEBQ), (55), translated into Chilean Spanish and validated for a Chilean population by Santos et al. (56). The instrument comprises 8 dimensions: 4 reflecting food approach behaviors and 4 reflecting food avoidance behaviors, scored on a 5-point Likert scale (1 = never, 5 = always). It has been used in studies in Chile and internationally (56–58). Parents or guardians will complete it at a parent meeting or online (estimated time: 10 min).

#### 4.2.4 Nutritional status and cardiometabolic risk

Anthropometric assessment will include height, body weight, and waist circumference (WC) using standardized school measurement protocols. Height will be measured with a portable SECA® stadiometer (model 217, Hamburg, Germany); body weight with a SECA® digital floor scale (model 876, Hamburg, Germany); and WC with a SECA® ergonomic tape measure (model 201, Hamburg, Germany) at the midpoint between the last rib and the iliac crest, placed horizontally without skin compression.

Assessment will be conducted by supervised, pre-trained nutrition and dietetics students in the school yard or gymnasium, accompanied by the kindergarten teacher or paraprofessional, with an estimated duration of 10 min per child (59). BMI-for-age will be calculated to classify nutritional status for preschoolers aged ≥5 years 1 month; WC will be classified using the WC-for-age (WC/A) indicator based on WHO growth standards adapted by Chile’s Ministry of Health (60). The waist-to-height ratio (WHtR) will be calculated to evaluate central obesity and cardiometabolic risk, using the evidence-based cut-off of ≥0.5. WHtR has been reported as a more efficient indicator than BMI for identifying metabolic risk in school-age children (61).

#### 4.2.5 Teachers’ perceptions of the active break program

Fourteen preschool teachers responsible for program implementation will be interviewed using a semi-structured interview design grounded in an interpretive phenomenological paradigm (62, 63). The objective is to capture teachers’ lived experience of implementation, including professional learning, organizational facilitators and barriers, and strategies developed to promote inclusion and address classroom diversity. Physical exercise has been shown to act as a catalyst for emotional well-being and sense of belonging (64, 65), making teacher perceptions particularly relevant for understanding program implementation and impact on students with SEN. Interviews will be audio-recorded in full (66).

#### 4.2.6 Sociodemographic data

A custom 33-item sociodemographic form (Supplementary material 5) will be completed by parents or guardians at baseline, collecting data on: student characteristics (name, date of birth, age, sex, city of residence, country of birth), SEN status, PIE participation, school schedule, extracurricular sports participation, home-school distance, transportation mode, proximity to public play spaces, school absences, medical conditions, vision, and hearing; and parental education level and PA frequency. This information will contextualize results and allow identification of patterns and between-group differences. Estimated completion time: 10 min.

## 5 Statistical analysis

Table 5 provides a summary of the statistical analysis plan.

**Table 5:** Statistical analysis plan.

Analysis	Variables	Method	Software
Descriptive	All continuous and categorical variables	Means, SD, medians, IQR; absolute/relative frequencies	JASP/R
Missing data	All outcome variables	Multiple imputation under intention-to-treat principle (67)	JASP/R
Normality	Continuous outcomes	Shapiro–Wilk test + Q-Q plot inspection	JASP
Homogeneity of variances	Continuous outcomes	Levene’s test	JASP
Primary inferential analysis	Motor competence; physical fitness	Linear mixed-effects models with school as a random effect and participant-level repeated measures; fixed effects: Group, Time, and Group × Time; Bonferroni-adjusted post-hoc comparisons; effect sizes: η_p_² or standardized beta estimates, as applicable.Same linear mixed-effects modeling framework; appropriate transformations or non-parametric alternatives if assumptions are violated.	JASP/R
Secondary outcomes	Learning outcomes, sleep, eating behavior, anthropometry	Same mixed ANOVA framework; non-parametric alternatives (Friedman/Kruskal-Wallis) if normality violated	JASP
Qualitative analysis	Teacher interview data	Content analysis with open coding (70); constant comparative method (72); researcher triangulation (73)	ATLAS.ti
Mixed-methods integration	All components	Connecting strategy (74): quantitative results inform qualitative interview construction	Narrative synthesis

### 5.1 Quantitative analysis

Data cleaning will include identification of missing values, detection of outliers, and verification of statistical assumptions. Missing data will be managed through multiple imputation under the intention-to-treat principle (67). Normality of residuals will be verified using the Shapiro–Wilk test and *Q*-Q plot inspection; homogeneity of variances will be assessed using Levene’s test. In the event of significant deviations from normality, appropriate transformations or non-parametric alternatives (Friedman or Kruskal-Wallis) will be considered.

A descriptive analysis will be conducted using measures of central tendency and dispersion for continuous variables, and absolute and relative frequencies for categorical variables. To account for the clustered nature of the study design and repeated observations over time, intervention effects will be analyzed using linear mixed-effects models. School will be included as a random effect to account for intra-cluster correlation, while participant-level repeated measures will be modeled across time. Fixed effects will include Group (TEG, PEG, CG), Time (baseline/post-intervention), and the Group × Time interaction. Post-hoc comparisons with Bonferroni correction will be conducted when appropriate. Effect sizes will be reported as partial eta squared (η^2^p) or standardized beta estimates where applicable, interpreted as: ≥0.01 small; ≥0.06 moderate; ≥0.14 large (68). All quantitative analyses will be conducted in JASP and/or R (*p* < 0.05); results will be visualized in Grapher.

### 5.2 Qualitative analysis

Qualitative data from audio-recorded teacher interviews will be transcribed and imported into ATLAS.ti (69). Content analysis with an open coding system will be applied (70), using systematic searches for emerging patterns (71) and constant comparative methods to identify the most frequently referenced characteristics within each theme across all cases (72). Researcher triangulation will ensure validity, depth, and breadth of results (73).

### 5.3 Mixed-methods integration

Integration is achieved through a connecting strategy (74): statistical results from the quantitative phase will inform the construction of interview guides for the qualitative phase, enabling explanation of organizational facilitators and barriers. The qualitative phase is grounded in Van Manen's (63) pedagogical phenomenology, capturing the lived experience of 14 preschool teachers.

## 6 Ethical and legal aspects

This study will be conducted in accordance with the ethical principles of the Declaration of Helsinki (75) and the Ethical Standards in Sport and Exercise Science Research (76). The protocol was evaluated and approved by the Scientific Ethics Committee of Universidad Santo Tomás: Approval Act CEC Res. N° 23,136,643/2023, dated March 24, 2026. The study complies with Chilean Law 20,120 on scientific research involving human subjects. All collected information will be safeguarded and stored by the principal investigators (MV-P and VG), ensuring participant confidentiality and exclusive use of data for academic purposes.

## 7 Discussion

This protocol makes a novel contribution by proposing one of the first controlled trials to simultaneously evaluate the efficacy of curriculum-integrated active breaks on motor competence, physical fitness, learning outcomes, and healthy habits in the preschool population. While consistent evidence supports the benefits of PA in the school context, this evidence is concentrated predominantly in primary school-age children; evidence for early childhood education, particularly for children aged 5–6 years, remains scarce (9, 31). This limitation is especially significant given that more than 80% of children globally do not meet international PA recommendations (3, 5)—a priority public health challenge that warrants targeted early intervention.

The study’s key contribution lies in the systematic integration of PA with curricular content through structured active breaks, moving beyond traditional approaches that treat PA as peripheral to education. This aligns with the Active Schools model (14, 15) and with embodied cognition theory, which posits that cognitive processes are intrinsically linked to motor action, potentially explaining the positive effects of PA on executive functions and academic performance (22, 24). While most studies demonstrating such effects have been conducted in primary education, the preschool years—a period of critical neuromotor and cognitive development—may amplify these benefits (10), underscoring the value of generating specific evidence at this developmental stage.

A substantive contribution is the protocol’s inclusive design, guaranteeing participation of students with and without SEN. Children with SEN present lower PA levels and greater participation barriers in school contexts, deepening health and learning inequities (23). The incorporation of adapted exercises, cooperative learning strategies, and interactive digital resources represents a relevant innovation aligned with inclusive education principles (13) and contributes evidence in an underexplored area.

From a public health and educational policy perspective, the evaluation of a feasible, low-cost intervention implementable by generalist classroom teachers addresses key scalability requirements. The Clases Activas digital platform adds value by facilitating standardization, monitoring, and sustainability of the program, addressing previously identified challenges in school-based implementation (28). This is particularly relevant in the Chilean context, where recent normative frameworks have begun to promote daily PA in schools.

The sequential explanatory mixed-methods design (DEXPLIS) significantly strengthens this protocol, enabling both evaluation of intervention efficacy and understanding of implementation mechanisms. The qualitative phase, grounded in Van Manen (63) pedagogical phenomenology, will help unpack the “black box” of school interventions by capturing teachers’ lived experiences—identifying barriers, facilitators, and contextual conditions that influence effectiveness (74). This approach is consistent with current recommendations for complex intervention studies in educational and public health settings.

Limitations should be acknowledged. The non-randomized design may introduce selection bias, limiting causal inference, although multiple imputation under intention-to-treat principles and cluster-adjusted analysis will mitigate this effect (67). The DIA EP, while nationally standardized, was designed primarily as a formative tool rather than for comparative research purposes, which constrains the interpretation of learning outcome comparisons between groups. Teacher- and family-reported measures in some secondary outcomes may also be subject to information bias. Future studies should consider randomized designs, objective PA measurement, and longer follow-up periods to strengthen causal evidence.

In conclusion, this protocol has the potential to generate novel and highly relevant evidence on the efficacy of active breaks in early childhood education. The curricular integration of PA, its inclusive and technology-supported design, and its mixed-methods framework position this trial as a promising strategy to simultaneously improve health, learning, and equity in the earliest stages of formal education. Findings may inform evidence-based policy and educational practice, contributing to a priority challenge in global child health.

## Footnotes

1
https://clasesactivas.cl/
