# Supplementary material for: Effects of school-based active breaks on motor competence, physical fitness, academic outcomes, and health behaviors in Chilean preschoolers: protocol for a three-arm, non-randomized cluster-controlled mixed-methods trial
Source: Front Public Health. 2026 Sep 23;14:1895830. doi: 10.3389/fpubh.2026.1895830 (PMC13642702; doi:10.3389/fpubh.2026.1895830)
Supplement: Supplementary file 1 [file Supplementary_file_1.docx]

**Supplementary Material**

Effects of school-based active breaks on motor competence, physical fitness, academic outcomes, and health behaviors in Chilean preschoolers: protocol for a three-arm, non-randomized cluster-controlled mixed-methods trial

**Article ID 1895830**

Maria Alejandra Valencia-Pacheco et al.

This consolidated document contains all supplementary materials cited in the article.

## Contents

- Supplementary Material 1 Schedule of enrollment intervention and assessments
- Supplementary Material 2 Safety procedures for active breaks
- Supplementary Material 3a Examples of active break questions and responses
- Supplementary Material 3b Distribution of curricular core components across 16 intervention weeks
- Supplementary Material 4 Evaluator training procedures
- Supplementary Material 5 Sociodemographic data form

# Supplementary Material 1

## Schedule of enrollment intervention and assessments

The study includes recruitment and baseline assessment, a 16-week active intervention phase conducted within calendar weeks 5-22, post-intervention assessment, and delayed delivery of the program to the waitlist control group.

| **Study activity** | **Recruitment and preparation** | **Baseline weeks 1-4** | **Intervention weeks 5-22** | **Post-test weeks 23-26** | **Post-trial** |
| --- | --- | --- | --- | --- | --- |
| School eligibility screening and institutional authorization | X |  |  |  |  |
| Parental or guardian informed consent | X |  |  |  |  |
| Child assent |  | X |  |  |  |
| Non-randomized school allocation to TEG PEG or CG |  | X |  |  |  |
| Baseline outcome assessment |  | X |  |  |  |
| TEG 10 active breaks per week |  |  | X |  |  |
| PEG 3-5 active breaks per week |  |  | X |  |  |
| CG regular school routine as waitlist |  |  | X |  |  |
| Post-intervention outcome assessment |  |  |  | X |  |
| Post-intervention teacher interviews |  |  |  | X |  |
| Active break program offered to the CG |  |  |  |  | X |

*TEG = Total Experimental Group; PEG = Partial Experimental Group; CG = Control Group. Outcome assessments included motor competence, health-related physical fitness, learning outcomes, sleep, eating behavior, and anthropometric indicators. The planned winter break during calendar weeks 13-14 was excluded from active intervention delivery.*

# Supplementary Material 2

## Safety procedures for active breaks

The following preventive measures will be implemented before and during each active break to minimize risks for preschool children.

## Physical space

- Remove unstable furniture, sharp objects, and any materials that could cause falls.
- Confirm that the floor is clean and dry.
- Ensure sufficient space for children to move safely by relocating chairs and tables when necessary.

## Equipment and digital resources

- Review audio and visual materials before the session to avoid confusing or distracting stimuli.
- Confirm that audio equipment is working correctly and set to an appropriate volume.
- Check that the platform is displayed clearly on the digital board or projector.

## Understanding the instructions

- Verify that children understand the instructions before beginning each exercise.
- Use simple language and visual examples.
- Demonstrate each exercise before children perform it.
- Check understanding through brief questions.

## Safety monitoring and incident response

- Continuously supervise the activity and identify potential incidents.
- Maintain a safety observation log and record every accident or risk situation.
- Adjust procedures in response to observations and accumulated implementation experience.
- If a child falls or is injured, the adults responsible for the class will provide immediate assistance and follow the school's established incident procedures.

## Hydration

Children will be encouraged to drink water before and after each active break.

# Supplementary Material 3a

## Examples of active break questions and responses

*Activities were delivered in Spanish. English translations are provided below. For the phonological tasks, Spanish stimulus words are retained because translation would change the target initial or final sound.*

## Interaction and understanding of the environment

**Core component Mathematical thinking**

Learning Objective 5 Orient themselves in time in everyday situations, using concepts and relationships of sequence (before, now, after, and at the same time; day and night), frequency (always, sometimes, and never), and duration (long and short).

**Objective 5.1 Arrange everyday events in sequence before now and after.**

| **No** | **Question** | **Correct response** | **Incorrect response** |
| --- | --- | --- | --- |
| 1 | What do you do after waking up? | Get up | Sleep |
| 2 | What do you do after lunch? | Play | Eat breakfast |
| 3 | What do you do after brushing your teeth? | Rinse your mouth | Eat |
| 4 | What do you do before going to bed? | Put on pajamas | Run |
| 5 | What are you doing now? | Participate | Eat |
| 6 | What do you do while eating? | Chew | Sleep |
| 7 | What do you do while running? | Move | Get dressed |
| 8 | What do you do before eating? | Wash your hands | Go to bed |

## Integral communication

**Core component Verbal language**

Learning Objective 3 Discover, in play-based contexts, phonological attributes of familiar words, such as word counting, syllable segmentation and counting, and identification of initial and final sounds.

**Objective 3.2 Identify initial and final sounds in words.**

| **No** | **Question** | **Correct response** | **Incorrect response** |
| --- | --- | --- | --- |
| 1 | What is the initial sound of the Spanish word "mesa" ("table")? | M | A |
| 2 | What is the final sound of the Spanish word "gato" ("cat")? | O | T |
| 3 | What is the final sound of the Spanish word "sol" ("sun")? | L | S |
| 4 | What is the initial sound of the Spanish word "pelota" ("ball")? | P | T |
| 5 | What is the final sound of the Spanish word "casa" ("house")? | A | C |
| 6 | What is the final sound of the Spanish word "pan" ("bread")? | N | P |
| 7 | What is the initial sound of the Spanish word "luz" ("light")? | L | U |
| 8 | What is the final sound of the Spanish word "mar" ("sea")? | R | M |

# Supplementary Material 3b

## Distribution of curricular core components across 16 intervention weeks

Two curricular prompts were scheduled per school day. The three core components rotated in a repeating sequence. Week numbers refer to active intervention weeks and exclude the scheduled winter recess.

| **Week** | **Monday** | **Tuesday** | **Wednesday** | **Thursday** | **Friday** |
| --- | --- | --- | --- | --- | --- |
| 1 | 1 Mathematical thinking 2 Coexistence and citizenship | 1 Verbal language 2 Mathematical thinking | 1 Coexistence and citizenship 2 Verbal language | 1 Mathematical thinking 2 Coexistence and citizenship | 1 Verbal language 2 Mathematical thinking |
| 2 | 1 Verbal language 2 Mathematical thinking | 1 Coexistence and citizenship 2 Verbal language | 1 Mathematical thinking 2 Coexistence and citizenship | 1 Verbal language 2 Mathematical thinking | 1 Coexistence and citizenship 2 Verbal language |
| 3 | 1 Coexistence and citizenship 2 Verbal language | 1 Mathematical thinking 2 Coexistence and citizenship | 1 Verbal language 2 Mathematical thinking | 1 Coexistence and citizenship 2 Verbal language | 1 Mathematical thinking 2 Coexistence and citizenship |
| 4 | 1 Mathematical thinking 2 Coexistence and citizenship | 1 Verbal language 2 Mathematical thinking | 1 Coexistence and citizenship 2 Verbal language | 1 Mathematical thinking 2 Coexistence and citizenship | 1 Verbal language 2 Mathematical thinking |
| 5 | 1 Verbal language 2 Mathematical thinking | 1 Coexistence and citizenship 2 Verbal language | 1 Mathematical thinking 2 Coexistence and citizenship | 1 Verbal language 2 Mathematical thinking | 1 Coexistence and citizenship 2 Verbal language |
| 6 | 1 Coexistence and citizenship 2 Verbal language | 1 Mathematical thinking 2 Coexistence and citizenship | 1 Verbal language 2 Mathematical thinking | 1 Coexistence and citizenship 2 Verbal language | 1 Mathematical thinking 2 Coexistence and citizenship |
| 7 | 1 Mathematical thinking 2 Coexistence and citizenship | 1 Verbal language 2 Mathematical thinking | 1 Coexistence and citizenship 2 Verbal language | 1 Mathematical thinking 2 Coexistence and citizenship | 1 Verbal language 2 Mathematical thinking |
| 8 | 1 Verbal language 2 Mathematical thinking | 1 Coexistence and citizenship 2 Verbal language | 1 Mathematical thinking 2 Coexistence and citizenship | 1 Verbal language 2 Mathematical thinking | 1 Coexistence and citizenship 2 Verbal language |
| 9 | 1 Coexistence and citizenship 2 Verbal language | 1 Mathematical thinking 2 Coexistence and citizenship | 1 Verbal language 2 Mathematical thinking | 1 Coexistence and citizenship 2 Verbal language | 1 Mathematical thinking 2 Coexistence and citizenship |
| 10 | 1 Mathematical thinking 2 Coexistence and citizenship | 1 Verbal language 2 Mathematical thinking | 1 Coexistence and citizenship 2 Verbal language | 1 Mathematical thinking 2 Coexistence and citizenship | 1 Verbal language 2 Mathematical thinking |
| 11 | 1 Verbal language 2 Mathematical thinking | 1 Coexistence and citizenship 2 Verbal language | 1 Mathematical thinking 2 Coexistence and citizenship | 1 Verbal language 2 Mathematical thinking | 1 Coexistence and citizenship 2 Verbal language |
| 12 | 1 Coexistence and citizenship 2 Verbal language | 1 Mathematical thinking 2 Coexistence and citizenship | 1 Verbal language 2 Mathematical thinking | 1 Coexistence and citizenship 2 Verbal language | 1 Mathematical thinking 2 Coexistence and citizenship |
| 13 | 1 Mathematical thinking 2 Coexistence and citizenship | 1 Verbal language 2 Mathematical thinking | 1 Coexistence and citizenship 2 Verbal language | 1 Mathematical thinking 2 Coexistence and citizenship | 1 Verbal language 2 Mathematical thinking |
| 14 | 1 Verbal language 2 Mathematical thinking | 1 Coexistence and citizenship 2 Verbal language | 1 Mathematical thinking 2 Coexistence and citizenship | 1 Verbal language 2 Mathematical thinking | 1 Coexistence and citizenship 2 Verbal language |
| 15 | 1 Coexistence and citizenship 2 Verbal language | 1 Mathematical thinking 2 Coexistence and citizenship | 1 Verbal language 2 Mathematical thinking | 1 Coexistence and citizenship 2 Verbal language | 1 Mathematical thinking 2 Coexistence and citizenship |
| 16 | 1 Mathematical thinking 2 Coexistence and citizenship | 1 Verbal language 2 Mathematical thinking | 1 Coexistence and citizenship 2 Verbal language | 1 Mathematical thinking 2 Coexistence and citizenship | 1 Verbal language 2 Mathematical thinking |

# Supplementary Material 4

## Evaluator training procedures

The evaluator training process included the following actions before school-based data collection.

**1.** Authorization was requested from program directors in Kinesiology, Nutrition and Dietetics, and Special Education for fourth- and fifth-year thesis students to participate in the preschool active break project.

**2.** Authorization and support were requested from the students' thesis supervisors so that the students could join the evaluator team.

**3.** A secure roster was prepared with the information required to organize all prospective evaluators.

**4.** Students were invited by email to the first evaluator team meeting.

**5.** The first meeting introduced the team and study, explained the active break program, reviewed a project video, included completion of an active break using the Clases Activas platform, provided an overview of MOBAK-KG and PREFIT, reviewed materials and organization, and verified evaluator details in the roster.

**6.** MOBAK-KG and PREFIT materials and training videos were emailed to evaluators for independent study. Availability was collected to schedule the second meeting.

**7.** Each evaluator recorded and submitted two brief self-simulation videos. The evaluator demonstrated one MOBAK-KG station and one PREFIT station for approximately two minutes each so that technique could be reviewed.

**8.** Each evaluator completed a 20-item online knowledge questionnaire covering both instruments.

Training questionnaire: https://docs.google.com/forms/d/e/1FAIpQLSfcdz8BDRcWrFAiewmQoFjfU1mpb32dYmstFvm9aNGK9xkNrw/viewform

**9.** The second meeting included a group simulation of both instruments and practical guidance for assessing children aged 5-6 years.

- Present tests as enjoyable games or challenges.
- Explain in simple language that activities are intended to show how children move and grow rather than to identify winners and losers.
- Prepare the child for assessment in the presence of a trusted adult to protect emotional well-being and reduce discomfort or embarrassment.
- Use positive reinforcement and emphasize effort rather than performance.
- Communicate calmly and supportively and avoid explicit comparisons between children.
- Observe emotional state directly to identify frustration and respond preventively.
- Review child assent, data recording forms, and risk prevention measures; assemble all testing stations and practice material use under the guidance of two trainers.

**10.** Evaluator schedules were organized for attendance at participating schools.

**11.** Before school visits, evaluators submitted the documents required by participating schools, including a criminal record certificate, a certificate confirming eligibility to work with minors, and a certificate concerning relevant maltreatment offenses.

# Supplementary Material 5

## Sociodemographic data form

*This custom 33-item form was completed by a parent, guardian, or legal representative at baseline. It was administered in Spanish; the English translation below is provided for international readers. The form is reproduced without participant responses.*

**Respondent information**

Name of person completing the form ________________________________________________

Relationship to the child ☐ Father	☐ Mother	☐ Other family member excluding parents	☐ Legal guardian

Child's name ___________________________________________________________________

Class or grade ____________________

School _________________________________________________________________________

Assessment date ______ / ______ / 2026

## Section A General child information

**1. Child's age in years**

Response: __________________________________________________________

**2. Date of birth**

Response: __________________________________________________________

**3. City of residence**

Response: __________________________________________________________

**4. Email address of parent or guardian**

Response: __________________________________________________________

## Section B Child characteristics

*This section characterizes the participating child.*

**5. Child's sex**

☐ Male

☐ Female

☐ Prefer not to state

**6. In which country was the child born?**

☐ Chile

☐ Other

Response: __________________________________________________________

**7. Child's place of residence**

☐ Urban

☐ Rural

**8. Which school schedule does the child attend?**

☐ Morning

☐ Afternoon

☐ Full school day

**9. Does the child participate in an extracurricular sport activity?**

☐ Yes

☐ No

**10. Approximately how far is the school from the child's home?**

☐ 1-5 city blocks

☐ 6-10 city blocks

☐ 11-15 city blocks

☐ More than 15 city blocks

**11. How does the child usually travel to and from school?**

☐ Walking

☐ Bicycle or scooter

☐ School van, shared taxi, car, or bus

**12. What clothing does the child usually wear to school?**

☐ School tracksuit

☐ School uniform

☐ Any type of clothing

**13. How often was the child absent from school during the previous year?**

☐ Very frequently more than 5 days per month

☐ Frequently 4-5 days per month

☐ Sometimes 2-3 days per month

☐ Rarely 1 day per month

☐ Never

**14. Does the child have temporary or permanent special educational needs?** *If yes state the diagnosis.*

☐ Yes

☐ No

Response: __________________________________________________________

**15. Does the child participate in the School Integration Program (Programa de Integracion Escolar, PIE)?**

☐ Yes

☐ No

**16. Is the child currently receiving professional care?** *Select all that apply.*

☐ Psychologist

☐ Speech-language pathologist

☐ Occupational therapist

☐ Kinesiologist or physical therapist

☐ Neurologist

☐ Special education teacher

☐ Other

Response: __________________________________________________________

**17. Where does the child receive this professional care?**

☐ At school

☐ Privately outside school

**18. Does the child currently have curricular accommodations?** *Select all that apply.*

☐ Access accommodations such as classroom placement or adapted materials

☐ Learning objective accommodations such as prioritization or modification of content

☐ None

**19. Does the child currently have a health condition requiring medical or pharmacological care?** *If yes provide details.*

☐ Yes

☐ No

Response: __________________________________________________________

**20. Does the child take medication associated with a special educational needs diagnosis?** *Provide the medication name and administration time especially if it is taken during school hours.*

Response: __________________________________________________________

**21. Does the special educational needs diagnosis affect participation in physical education classes or recess?** *If yes explain why.*

☐ Yes

☐ No

Response: __________________________________________________________

## Section C Child vision hearing mobility and communication

*For vision questions use of glasses includes spectacles or contact lenses whether used continuously or occasionally.*

**22. Does the child use corrective lenses or contact lenses?**

☐ Yes

☐ No

**23. How would you rate the child's vision?** *If corrective lenses are used rate vision while using them.*

☐ Good

☐ Fair

☐ Poor

**24. Does the child hear normally in both ears?**

☐ Yes

☐ No

**25. Can the child follow a television program at a volume acceptable to other people?**

☐ Yes

☐ No

**26. Can the child follow a conversation involving three or more people?**

☐ Yes

☐ No

**27. Does the child use any assistive device for mobility or communication?** *Examples include a wheelchair hearing aids or augmentative communication devices. If yes specify.*

☐ Yes

☐ No

Response: __________________________________________________________

## Section D Parent or guardian characteristics

*This section characterizes the child's parents guardians or legal representatives.*

**28. What is the highest level of education completed by the father?**

☐ None

☐ Primary education

☐ Secondary education

☐ Higher education

**29. What is the highest level of education completed by the mother?**

☐ None

☐ Primary education

☐ Secondary education

☐ Higher education

**30. Does the father regularly participate in physical activity or sport?**

☐ Yes once per week

☐ Yes three times per week

☐ Does not participate in physical activity

**31. Does the mother regularly participate in physical activity or sport?**

☐ Yes once per week

☐ Yes three times per week

☐ Does not participate in physical activity

**32. Is there a park, sports court, or gym near the home where the child can play?**

☐ Yes

☐ No

**33. If there is a park, sports court, or gym near the home, how often does the child use it?**

☐ Never

☐ Very rarely (once per week)

☐ Sometimes (three times per week)

☐ Frequently (five times per week)

☐ Always (seven times per week)
